# Cyanobacteria-Fungi Co-Cultures: Which Partner Contributes to Antifungal Activity?

**DOI:** 10.1007/s00284-024-03914-3

**Published:** 2024-10-10

**Authors:** Wendy A. Stirk, Bernadett Pap, Gergely Maróti, Johannes van Staden, Vince Ördög

**Affiliations:** 1 Research Centre for Plant Growth and Development, School of Life Sciences, University of KwaZulu-Natal Pietermaritzburg Campus, P/Bag X01, Scottsville, Pietermaritzburg, 3209 South Africa; 2 Institute of Plant Biology, HUN-REN Biological Research Centre, Temesvári Krt, 62, 6726 Szeged, Hungary; 3 Faculty of Water Sciences, Ludovika University of Public Service, 6500 Baja, Hungary; 4 Department of Plant Sciences, Faculty of Agricultural and Food Sciences, Széchenyi István University, Kolbai K. Str 8, 9200 Mosonmagyaróvár, Hungary

## Abstract

Cyanobacteria synthesize secondary metabolites with antifungal activity, making them potential biopesticide agents for sustainable, eco-friendly agriculture. Programmes to identify Cyanobacterial strains with effective bioactivity generally screen strains maintained in culture collections. These strains are often monoclonal but non-axenic and this may potentially influence the bioactivity of the generated biomass. The present study investigated in vitro antifungal activity of *Nostoc muscorum* MACC-189 and *N. linckia* MACC-612 strains co-isolated with fungal co-partners and maintained in the Mosonmagyaróvár Algal Culture Collection (MACC). The fungal co-partners were isolated from the Cyanobacterial stock cultures and identified as *Purpureocillium lilacinum* and *Sarocladium* sp., respectively. The cultures were tested against seven phytopathogens. The phytopathogenic fungi were grown on potato dextrose agar plates and suspension cultures of the Cyanobacteria-fungi and isolated fungal co-partners were placed in the centre of the plate. Antifungal effects were assessed semi-quantitatively after 10 days of incubation. The Cyanobacteria-fungal co-cultures had antifungal activity against *Monilinia fructigena* and *Aspergillus* sp. with the *N. muscorum*/*P. lilacinum* culture being the most effective. The fungal isolates inhibited *M. fructigena* with *P. lilacinum* having a dose-dependent response but did not inhibit *Aspergillus* sp. This suggested that the antifungal effect of the Cyanobacterial cultures on *M. fructigena* was due to the fungal partner rather than the cyanobacterium while the antifungal effect on *Aspergillus* sp. was due to the cyanobacterium partner. As it was not possible to maintain living axenic *N. muscorum* and *N. linckia* cultures, this could not be conclusively confirmed. These results highlight the importance of either using axenic cultures or identifying the co-isolates when testing Cyanobacteria cultures for antifungal bioactivity.

## Introduction

Culture collections provide a repository of living Cyanobacteria and microalgae strains collected from diverse geographic locations and unique ecological habitats [1]. They are a valuable resource for many fundamental and applied research projects needing access to diverse taxonomic strains [2] such as screening for antifungal activity. As it is difficult to isolate and maintain axenic cultures, many of the strains in these collections are monoclonal with a consortium of microorganisms living within their phycosphere. These microorganisms were either associated with algae in the environment where they were collected or due to contamination when routinely transferred to fresh medium [1–4]. The bacteria and fungi co-isolates may be dispersed in the media or embedded in the mucilage surrounding the algal cells where they form a close, often symbiotic association [5]. Thus, each algal strain in culture collections will have its own specific microorganism community. For example, analysis of 12 *Botryococcus braunii* cultures from different culture collections showed high microbial diversity with each culture having a specific bacterial community [6].

Synthetic agrochemicals have long-term detrimental effects on environment, human and soil health. Current research is focused on developing environmentally friendly natural products for sustainable agriculture [7]. This approach encompasses using biological control agents to manage soil-borne phytopathogens. The benefits of biopesticides include being easily biodegradable, having a lower mammalian toxicity than synthetic chemical agents and reducing the likelihood of fungicide-resistant strains developing [8].

Cyanobacteria synthesize diverse secondary metabolites with over 2000 secondary metabolites identified to date with many of these metabolites unique to the Cyanobacteria [9]. Their ecological role includes chemical defense against other organisms (antibacterial and antifungal activity), facilitating symbiosis, antioxidant activity and signalling molecules [10]. To date, 106 antifungal compounds (mainly peptides and alkaloids) have been identified in Cyanobacteria [11]. These antifungal compounds make Cyanobacteria potential biocontrol agents for managing plant diseases. Many Cyanobacteria have potent in vitro antifungal activity against phytopathogenic fungi [7]. For example, in vitro antifungal activity of water extracts of 19 of the 33 Cyanobacteria strains tested against six phytopathogenic fungi and three phytopathogenic oomycetes had antifungal activity against at least one phytopathogen. Five strains had a broader spectrum of activity, inhibiting more than three phytopathogens [12]. They are also effective antifungal agents *in planta* [7]. For example, *Nostoc calcicola* and *N. linckia* reduced the incidence of the tomato wilt pathogen *Fusarium oxysporum* f. sp. *lycopersici* and increased the yield of tomato fruits [13].

*Nostoc* species are heterocystous, filamentous Cyanobacteria. They produce large amounts of exopolysaccharides which form a dense sheath, often encasing the whole colony [14] which can stimulate the growth of contaminants. Many *Nostoc* species have antifungal activity and are a potential source of biopesticides [15]. Ideally, screening for bioactive compounds should be conducted using axenic cultures to ensure that the bioactivity is due to compounds synthesized by the Cyanobacteria rather than the consortium of co-isolates but this is often not the case. The aim of the present study was to identify the fungi co-living in two bioactive *Nostoc* strains and to establish if these microbes contributed to the bioactivity of the biomass.

## Materials and Methods

### Identification of Cyanobacteria Strains and Contaminating *Fungi*

Two Cyanobacteria *Nostoc muscorum* MACC-189 and *Nostoc linckia* MACC-612 from the Mosonmagyaróvár Algal Culture Collection (MACC) of the Széchenyi István University, Hungary were used. *N. muscorum* MACC-189 was isolated from a soil sample collected in Serbia in 1984. *N. linckia* MACC-612 was isolated from a water sample collected in the Czech Republic in 1984. Both strains were co-isolated with fungal co-partners. The cultures are maintained in solidified medium in low light at 15 ± 2 °C and subcultured every 6–8 months. The identity of the *Nostoc* strains was confirmed by 16S rDNA and 18S rDNA/ITS marker gene sequencing using algal genomic DNA purified from cultures based on single algal colonies [16]. The fungi were isolated from the two Cyanobacterial cultures and identified by 18S rDNA and ITS marker gene sequencing using fungal genomic DNA purification via the Chelex method [16]. The isolated fungi were maintained on potato dextrose agar (PDA) plates at 25 °C. It was not possible to eliminate the fungi partner from the Cyanobacteria. Thus, no living axenic *Nostoc* cultures could be maintained.

### Screening for Antifungal Activity

Suspensions of the Cyanobacteria-fungal and isolated fungi cultures were screened for in vitro antifungal activity. The Cyanobacteria from the solidified media were inoculated into two flasks containing 250 mL Zehnder-8 nutrient medium [17] and cultured for 24 days at 30 °C in a 16:8-h light:dark photoperiod and illuminated from below with 50 μmol photons/m^2^/ s light intensity. Cultures were aerated with 20 L/h sterile air enriched with 1.5% CO_2_ during the light period. The cultures were centrifuged (2000 × *g,* 10 min, 20 °C) and the wet weight of the pellet measured. The pellet was resuspended in sterile MilliQ water at 1 g FW/mL. Suspension cultures of the isolated fungi were prepared in sterile MilliQ water at 1 g FW/mL.

The prepared suspensions were tested against seven fungal strains. These included four phytopathogenic strains (*Alternaria alternata* AaHA, *Botrytis cinerea* BcHA, *Fusarium oxysporum* CBS 123668 and *Monilinia fructigena* MfHA), two human pathogens (*Aspergillus* sp. isolated from a soil sample collected in Hungary in 2022 and *Candida albicans* ATCC14053) and a brewer’s yeast strain *Saccharomyces cerevisiae* ATCC9763. Suspension cultures (1 g FW/mL) were prepared from the seven fungal strains and 100 µL spread on PDA plates (9-cm petri dishes) and the plates air-dried. Then 20 µL suspension culture of the Cyanobacteria-fungi and isolated fungi were placed in the centre of the plates. The plates were sealed with parafilm and incubated at room temperature for 10 days. Antifungal effects were assessed semi-quantitatively with the activity described as no activity (−); mild antifungal activity ( +) and stronger activity (+ +). Nystatin antifungal agent (10 U/mL) was used as a positive control and distilled water as a negative control. Three replicate plates were prepared.

### Effect of Fungal Suspension Culture Concentration on Antifungal Activity

The effect of fungal suspension concentration on antifungal activity was tested using *Purpureocillium lilacinum* and *Sarocladium* sp. suspension cultures against *M. fructigena*. For this, the three fungal cultures were grown in potato dextrose broth (PDB). These were centrifuged (2000 × *g*, 10 min, 20 °C). The wet weight of the pellet was measured and suspended at 1 g FW/mL in sterile MilliQ water. *M. fructigena* suspension (100 µL) was spread on a PDA plate and air-dried. *P. lilacinum* and *Sarocladium* sp. suspensions (10 µL) were placed in the centre of the plate. In addition, these suspensions were diluted (0.1, 0.01 and 0.001 g FW/mL) and 10 µL placed on the PDA plates. The plates were air-dried, sealed and incubated at room temperature for 10 days. The inhibition zone was irregular, and it was therefore estimated semi-quantitatively (− , + , + +). Three replicate plates were prepared.

## Results and Discussion

### Identification of Co-Isolates

Both 16S rDNA and 18S rDNA/ITS marker sequencing confirmed the identity of *Nostoc muscorum* MACC-189 and *Nostoc linckia* MACC-612. The fungus isolated from the *N. muscorum* culture was identified as *Purpureocillium lilacinum* (Hypocreales, Ophiocordycipitaceae; formerly *Paecilomyces lilacinus*). The fungus isolated from the *N. linckia* culture was identified as *Sarocladium* sp. (reallocated from *Acremonium* genus).

It was not possible to maintain living axenic *Nostoc* cultures once the fungal partner had been eliminated from the Cyanobacterial cultures for the two strains used in the present study. It is often difficult to get axenic cultures of Cyanobacteria as they naturally live in complex communities with microorganisms within or close to the exopolysaccharide layer [4]. While some interactions with the mycobiont may be antagonistic, other interactions are mutually beneficial [6, 18]. In these symbiotic interactions, the Cyanobacteria (photobiont) provide organic carbon while the heterotrophic mycobiont produces CO_2_, scavenges excess O_2_, generates growth-stimulating compounds (e.g. indole-3-acetic acid and vitamin B_12_) and can improve nutrient utilization via phosphate solubilization and siderophore production. The microbes also secrete antibiotics which improve the host’s defence and increase the rate of organic product degradation [18]. This enhances the fitness of the Cyanobacteria and enhances community resilience to invasion by other microbial species [6]. Thus, non-axenic cultures often grow better than axenic cultures under laboratory conditions [19]. The mutualistic relationship between co-partners may change (e.g. from facultative to obligatory) over time when strains are maintained in culture [18] as may be the case in the present study as the isolates have been in culture for 40 years (collected in 1984). Further studies are necessary to investigate potential mutualistic effects including more robust Cyanobacterial growth in the presence of a fungal co-partner.

### Antifungal Activity

**Fig. 1:**
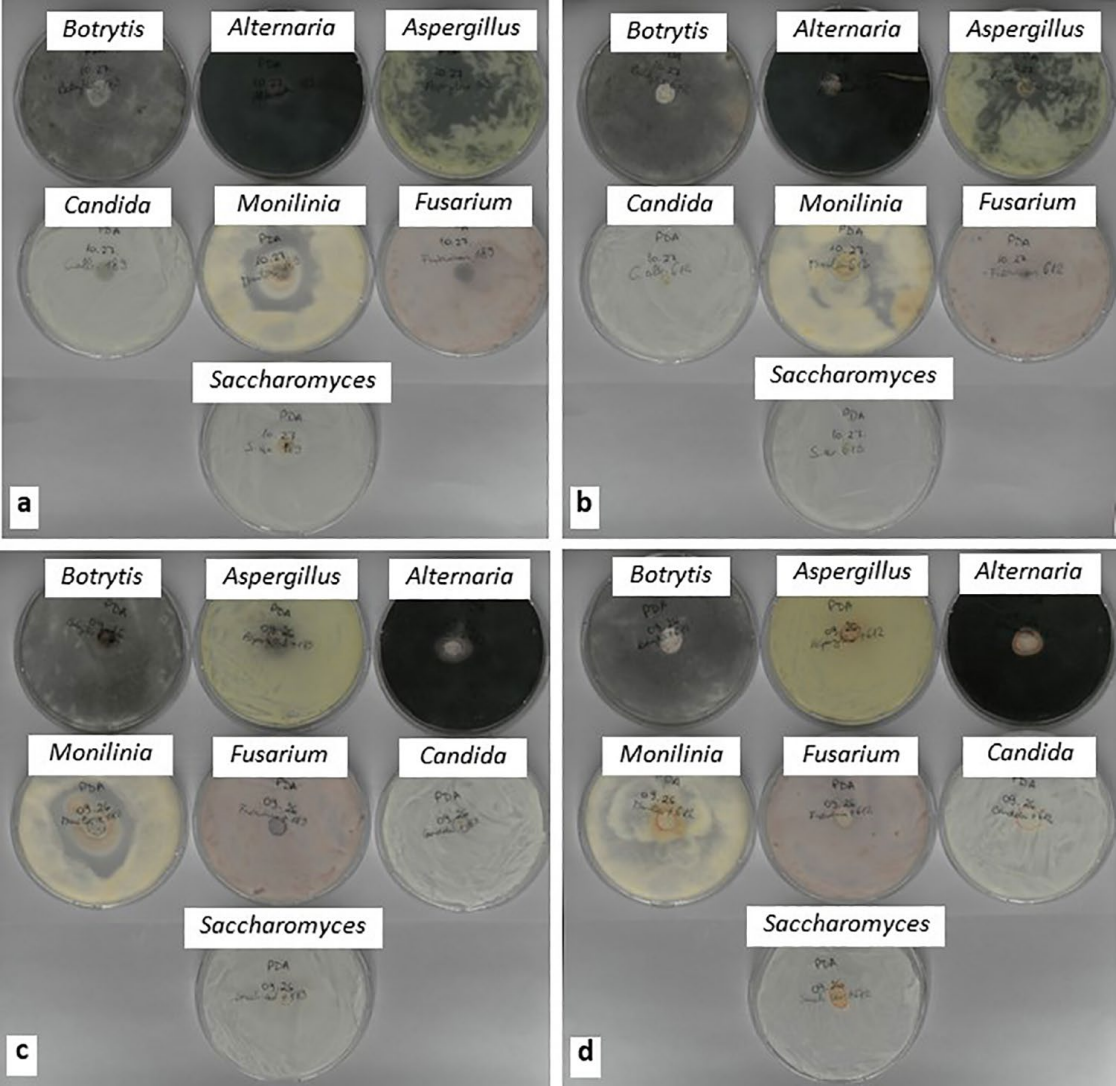
Representative plates showing antifungal activity of **a** *Nostoc muscorum-Purpureocillium lilacinum* co-culture; **b** *Nostoc linckia-Sarocladium* sp. co-culture; **c** *Purpureocillium lilacinum* isolated from *Nostoc muscorum*; and **d** *Sarocladium* sp. isolated from *Nostoc linckia* tested against seven fungal strains

The *N. muscorum* MACC-189*/P. lilacinum* culture exhibited antifungal activity against *M. fructigena* and *Aspergillus* sp. but had no effect on the other five fungal strains (Table 1; Fig. 1a). The *N. linckia* MACC-612*/Sarocladium* sp. culture also had an inhibitory effect on *M. fructigena* and *Aspergillus* sp. although the bioactivity was milder (Table 1; Fig. 1b). These two strains were selected for the present study based on a screening study where water extracts of *N. linckia* MACC-612 had in vitro fungicidal activity against four phytopathogens and fungistatic activity against three phytopathogens. *N. muscorum* MACC-189 had antifungal activity against four phytopathogens [12].

**Table 1:** In vitro antifungal activity in Cyanobacteria-fungi co-cultures and fungal isolates tested against seven pathogenic fungal strains

	Fungal strains						
	*Alternaria alternata*	*Botrytis cinerea*	*Monilinia fructigena*	*Fusarium oxysporum*	*Aspergillus* sp.	*Candida albicans*	*Saccharomyces cerevisiae*
Co-cultures (1 g FW/mL)							
*N. muscorum* + *P. lilacinum*	−	−	+ +	−	+	−	−
*N. linckia* + *Sarocladium* sp.	−	−	+	−	+	−	−
Fungal isolates (1 g FW/mL)							
*P. lilacinum*	−	−	+ +	−	−	−	−
*Sarocladium* sp.	−	−	+	−	−	−	−
Standards							
Distilled water	−	−	−	−	−	−	−
Nystatin (10 U/mL)	+ +	+ +	+ +	+ +	+ +	+ +	+ +

Results are presented semi-quantitatively as no activity ( −), mild activity ( +) and stronger activity (+ +). There were three replicate plates per strain

**Fig. 2:**
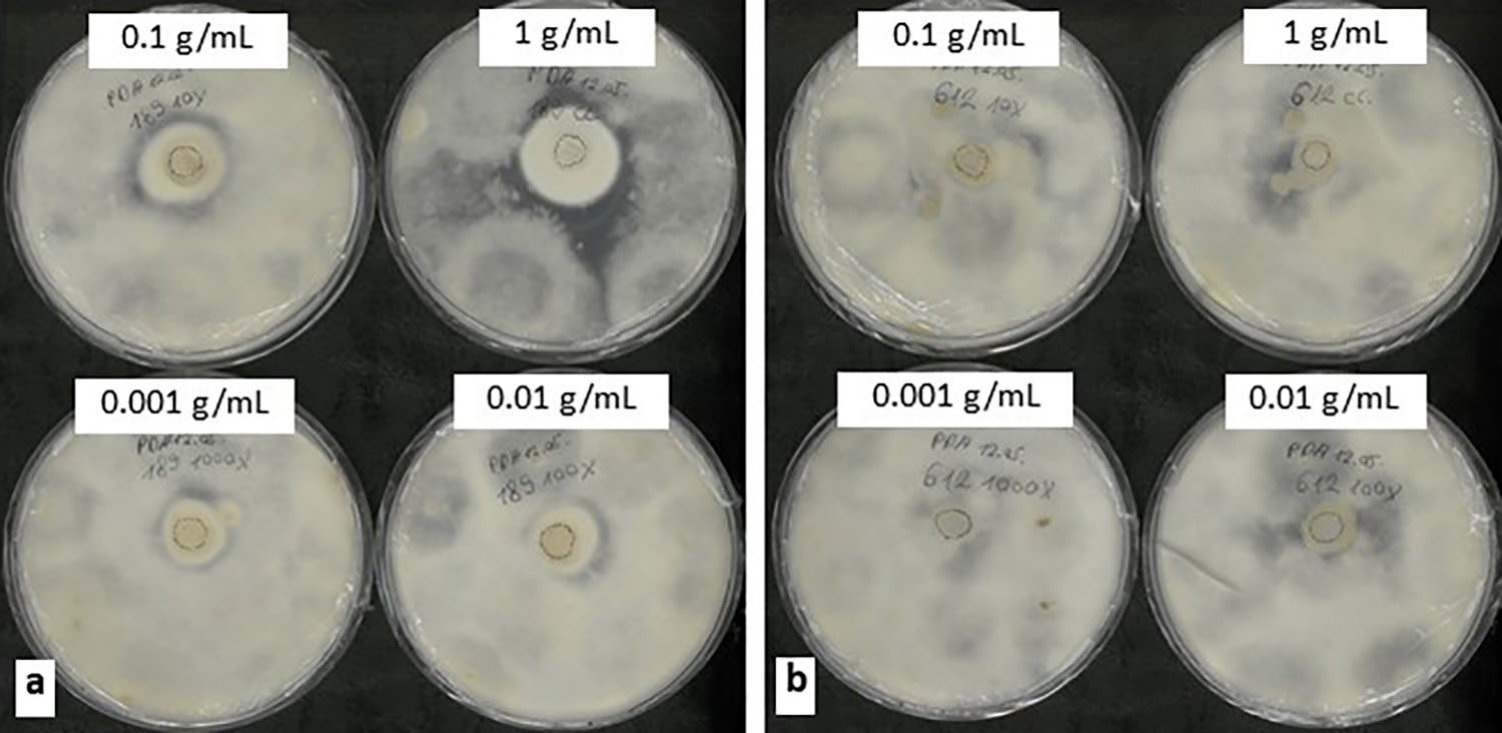
Representative plates showing antifungal activity of **a** *Purpureocillium lilacinum* isolated from *Nostoc muscorum*; and **b** *Sarocladium* sp. isolated from *Nostoc linckia* tested against *Monilinia fructigena* where the suspensions were applied at concentrations ranging from 1.0 to 0.001 g FW/mL

The isolated *P. lilacinum* suspension inhibited the growth of *M. fructigena* with similar levels of activity to the Cyanobacteria-fungi cultures but had no effect on the other six fungal strains, including *Aspergillus* sp. (Table 1; Fig. 1c). This activity was consistent for all replicate plates. Undiluted *P. lilacinum* suspension inhibited *M. fructigena* growth with a dose-dependent effect where the more dilute suspensions were less effective (Table 2; Fig. 2a). This indicated that the antifungal activity against *M. fructigena* in the non-axenic *Nostoc* cultures was mainly due to the fungal co-partner rather than the Cyanobacterial partner. In contrast, *Aspergillus* sp. was inhibited by the co-cultures but not the isolated fungi, suggesting that antifungal activity against *Aspergillus* sp. was due to the Cyanobacteria co-partner. As it was not possible to maintain living axenic *Nostoc* cultures in the present study, it could not be conclusively confirmed that the in vitro antifungal activity against *M. fructigena* and *Aspergillus* sp. were specific to the fungi and Cyanobacteria co-partners, respectively. Further investigation on the bioactivity of axenic Cyanobacterial strains, fungal isolates and co-cultures are required to established potential synergistic effects between the co-partners.

**Table 2:** In vitro antifungal activity of the two fungi isolated from the Cyanobacteria-fungi co-cultures tested in a range of concentrations against *Monilinia fructigena*

	Fungal isolates	
	*P. lilacinum*	*Sarocladium* sp.
1 g FW/mL	+ +	+
0.1 g FW/mL	+	+
0.01 g FW/mL	+	+
0.001 g FW/mL	−	−
Standards		
Distilled water	−	−
Nystatin (10 U/mL)	+ +	+ +

Results are presented semi-quantitatively as no activity (−), mild activity ( +) and stronger activity (+ +). There were three replicate plates per strain

The isolated *Sarocladium* sp. suspension only slightly inhibited the growth of *M. fructigena* (Table 1; Fig. 1d) and there was no concentration-dependent effect (Table 2; Fig. 2b). This activity was consistent for all replicates. Three volatile antifungals that were effective at inhibiting *Fusarium oxysporum* f. sp. *cubense* (wilt disease) have been identified in *Sarocladium brachiariae* [8].

It is possible to keep the co-isolate (bacteria and fungi) population low by using algal-specific media when non-axenic Cyanobacteria are grown in photoautotrophic conditions in the laboratory. This favours microalgae growth and minimizes the co-isolates’ growth so that they do not outcompete the target strain [3]. However, scaling up for industrial applications often requires growth under mixotrophic conditions. In these conditions, the microorganism within the phycosphere can utilize the carbon source so that the generated biomass is not due to Cyanobacterial growth alone. Apart from affecting the biomass dry weight, the co-isolates may also affect the concentration of bioactive compounds and hence biological activity of the biomass [20] as would occur in the *N. muscorum*/*P. lilacinum* co-culture where both partners have antifungal activity against different pathogens.

Constructing an artificial consortium of mutualistic microorganisms improves culture productivity by mimicking nature [18]. The co-cultures are more robust, stable and self-resilient when scaled up [19, 21] with reduced contamination by external microorganisms [22]. The results of the present study suggest a possible mutualistic relationship between *N. muscorum* and *P. lilacinum*. These compatible strains could potentially be co-cultured to produce living biomass for application as a combined biofertilizer and biopesticide product to improve soil health, crop productivity and disease resistance. *P. lilacinum* is a cosmopolitan, endophytic fungus commonly found in soil and rhizosphere of many plants [23]. It is a promising biocontrol agent with bioactivity against a range of disease-causing pathogens including antifungal activity [24, 25]. It is also an effective biocontrol agent against root-knot nematodes [26] and has insecticidal activity [23]. It has other beneficial traits (indole-3-acetic acid production, ammonia production, phosphate solubilization and chitinase production) that contribute to improved plant growth and increased availability of N and P in the soil [24, 27]. This makes *P. lilacinum* an attractive potential biocontrol agent with multiple plant growth-promoting traits. Many *Nostoc* species are able to withstand desiccation, freezing, high-UV and high-salinity conditions with metabolic processes recovering rapidly after rehydration [14]. These characteristics make then potentially suitable to develop as “fungi-carriers” for agriculture, with dual functions to protect the beneficial microorganisms as well as contributing to soil fertility and plant growth. Living biomass produced by co-culturing fungi and Cyanobacteria could potentially infer more resilience to the fungi under field conditions so that they remain viable for longer and more tolerant to adverse abiotic conditions [23]. Biomass produced from co-cultures of *N. muscorum*/*P. lilacinum* will need to be investigated under field conditions to establish if they enhanced crop performance.

## Conclusions

The fungal co-partners of the monoclonal *N. muscorum* MACC-189 and *N. linckia* MACC-612 were identified as *P. lilacinum* and *Sarocladium* sp., respectively. Isolates of the fungal co-partners could be cultured but it was not possible to maintain axenic *Nostoc* cultures under laboratory conditions. This highlighted a possible mutualistic relationship between the Cyanobacteria and fungi co-partners. The co-cultures had in vitro antifungal activity against *M. fructigena* and *Aspergillus* sp. with the *N. muscorum*/*P. lilacinum* culture being the most effective. The fungal isolates inhibited *M. fructigena* with *P. lilacinum* having a dose-dependent response but did not inhibit *Aspergillus* sp. This indicated that the antifungal effect of the co-cultures on *M. fructigena* was due to the fungal partner and the antifungal effect on *Aspergillus* sp. was due to the Cyanobacterium partner. As it was not possible to maintain living axenic *N. muscorum* and *N. linckia* cultures, this could not be conclusively confirmed. These results highlight the importance of either using axenic cultures or identifying the co-isolates and their bioactivities when testing Cyanobacteria strains for antifungal or other biologically active compounds.

## Data Availability

All data generated during this study are included in this published article.
